# Assessing the burden of osteoporosis and clinical fragility fractures in the French general population: insights from linked healthcare claims and health interview survey data used for surveillance

**DOI:** 10.1007/s11657-025-01616-2

**Published:** 2025-10-27

**Authors:** Joël Coste, Laurence Mandereau-Bruno, Panayotis Constantinou, Tatjana T. Makovski, Laure Carcaillon-Bentata, Francis Guillemin

**Affiliations:** 1 Department of Non-Communicable Diseases and Injuries, French Public Health Agency (Santé Publique France), 12 Rue du Val d’Osne, Saint-Maurice, 94410 France; 2 Data Science Division, French Public Health Agency (Santé Publique France), Saint-Maurice, France; 3 Direction of Strategy, Studies and Statistics, French National Health Insurance (Cnam), Paris, France; 4 Bordeaux PharmacoEpi, INSERM CIC-P 1401, Université de Bordeaux, Bordeaux, France; 5 CHRU-Nancy, Université de Lorraine, CIC 1433 Epidémiologie Clinique, InsermNancy, France

**Keywords:** Osteoporosis, Fragility fracture, Assessment, Health interview survey, Heath claims database

## Abstract

**Summary:**

Healthcare claims and survey data are increasingly used to assess the osteoporosis burden, but agreement and comparative validity of derived indicators are poorly documented. We show that no single data source can estimate the osteoporosis burden. Instead, coupling data sources allows assessing its burden and associated treatment and knowledge gaps.

**Purpose:**

Healthcare claims data are increasingly used to assess the burden of osteoporosis and fragility fractures, although comparative evidence with other sources and especially self-reported data remains limited. Using the linkage of the French National Health Data System (SNDS) and Health Care and Insurance Survey (ESPS 2010-2014), we evaluated the agreement and comparative validity (concurrent and predictive) of several osteoporosis and clinical fragility fracture indicators and provided comprehensive estimates of their prevalence.

**Methods:**

Individual data from 5039 ESPS participants aged ≥ 25 years were linked to SNDS. Follow-up data included a health self-assessment in 2014 and 5-year occurrence of fractures and mortality. Prevalence was estimated for each indicator (self-reported in ESPS, diagnosis and treatment of osteoporosis, and clinical fragility fractures in SNDS) using several combinations and capture-recapture. Kappa statistics assessed agreement between indicators. Multivariate models evaluated determinants of disagreement between sources and associations of indicators with health outcomes and new fractures (concurrent and predictive validity).

**Results:**

Prevalence estimated by capture-recapture was 7.6% versus 4.1% and 2.2% for self-reported and treated osteoporosis, respectively. Agreement between indicators was slight to moderate. Education, limitation in daily activities, and number of chronic conditions influenced agreement. SNDS indicators had better validity than self-reported osteoporosis, especially for predicting new fractures.

**Conclusion:**

The French healthcare claims database provides valid indicators, although it is insufficient to assess and monitor the burden of osteoporosis in the general population. Coupling these indicators with self-reported data may help overcome these limitations and assess the treatment and knowledge gaps associated with osteoporosis.

**Supplementary Information:**

The online version contains supplementary material available at 10.1007/s11657-025-01616-2.

## Introduction

Osteoporosis represents a major public health challenge worldwide through its association with fragility or low-energy fractures [1]. However, considerable difficulties are encountered when assessing and monitoring its burden in the population. In line with the WHO definition of osteoporosis, which relies on bone mineral density (BMD) measurements (BMD T-score ≤  − 2.5), health examination surveys in numerous countries provide estimates of low bone mineral density (LBMD) [2, 3]. However, general-population health examination surveys are expensive, time-consuming, and potentially affected by participation biases [4]. Moreover, as LBMD is only one among several factors of bone strength and fracture risk, it may lack validity for epidemiological purposes [5–7]. Health interview surveys have also been used in several countries to assess self-reported osteoporosis [8–10]. These studies are easier to conduct and less expensive, although they may also suffer from imprecise questions and various misclassification errors [4]. Recently, healthcare claims data have been proposed to identify subjects diagnosed with and/or treated for osteoporosis [11–13] and clinical fragility fractures [11, 13–17]. Health claims studies have well-known advantages, notably their moderate cost and reliability, and when conducted at a nationwide scale, their population coverage and representativeness [18, 19]. However, comparative evidence of healthcare claims indicators with other indicators, especially self-reported data, remains limited for osteoporosis [20–25]. Such comparisons are useful when informing decisions regarding the use of data sources to provide reliable estimates of the burden of the condition. Taking advantage of the linkage of the French National Health Data System (SNDS) and the Health Care and Insurance Survey (ESPS 2010-2014), a longitudinal interview health survey, the present study aims to evaluate the agreement and comparative validity (concurrent and predictive) of osteoporosis and clinical fragility fracture indicators constructed independently in both data sources. This study also provides recent and more comprehensive estimates of the burden of osteoporosis and clinical fragility fractures in France.

## Methods

### Data sources

#### ESPS survey

ESPS was a longitudinal health interview survey representative of individuals living in households in France (95% of the total population). It collected a wide range of information regarding health conditions, determinants, and outcomes. The full methodology of this survey has already been described elsewhere [26]. In 2010, the participation rate was 65%, resulting in 14,875 participants aged ≥ 25 years, approximately half of whom (one per household) were scheduled for reassessment in 2014.

#### SNDS database

The SNDS comprehensively collects pseudo-anonymized individual healthcare consumption data reimbursed to the beneficiaries of the various French public health insurance schemes since 2006 (98% of the French population in 2010) [27]. The SNDS includes medication reimbursements, laboratory tests, paraclinical investigations (but not the results), inpatient medical information from public and private hospitals, and long-term chronic diseases (LTDs) eligible for 100% reimbursement of related healthcare expenditure. Medications are coded using the Anatomical Therapeutic Chemical (ATC) classification system, whereas medical and surgical procedures as well as imaging investigations are coded using a standardized coding system of clinical procedures known as the Classification Commune des Actes Médicaux (CCAM, i.e., Common Classification of Medical Procedures). LTDs and hospital diagnoses are coded using the International Classification of Diseases, 10th Revision (ICD-10), although the reasons for the outpatient consultations (diagnoses) are not coded by any public or private primary care or specialist physician, whether within the hospital or other facility setting. The date of death is also recorded in the SNDS.

#### Linkage procedure

Linkage of the ESPS with the SNDS was limited to beneficiaries of the general health insurance scheme (salaried or retired employees of the private sector) and local mutualist sections (active or retired civil servants, students, etc.) representing 87% (in 2010) of the French population for whom SNDS data have been complete and reliable since 2006. The linkage using the unique national register number (French National Data Protection consent N°1748452) was possible for 7112 participants (97.5%) of whom 5039 (71%) completed the entire 2010 health assessment questionnaires (Supplementary Fig. 1).

### Osteoporosis and clinical fragility fracture indicators

#### Self-reported osteoporosis (ESPS)

Osteoporosis was included among the list of 61 chronic or recurrent conditions recorded in ESPS 2010 through a question asked to participants: “What illness(es) or health problem(s) do you currently have?,” “Osteoporosis,” “Yes,” or “No.”

#### Diagnosed osteoporosis (SNDS)

Participants with diagnosed osteoporosis were identified by the ICD-10 M80-M82 codes used for LTD registration or hospital diagnoses within the 5 years preceding the 2010 assessment (i.e., from 2006 to 2010).

#### Treated osteoporosis (SNDS)

Participants with treated osteoporosis were identified with the ATC code M05B for drugs affecting bone structure and mineralization (DABSM). At least three reimbursements of a DABSM at three different dates within the past 12 months preceding the 2010 assessment were required (two dates in the case of a 90-day supply).

#### Clinical fragility fractures (SNDS)

In line with the literature and consistent with the exponential increase in fracture rates at all sites from 70 years onwards in men and 50 years onwards in women, as observed in France [16] and elsewhere [28], any fracture identified as occurring beyond these age thresholds within the 5 years preceding the 2010 assessment (i.e., from 2006 to 2010) was considered to indicate a “fragility fracture” (beyond the 50- and 70-year thresholds, high-energy fractures become negligible compared to low-energy fractures in women and men). Algorithms previously developed and used to identify incident clinical fractures in France were used [16]. These algorithms considered fractures as either hospitalized (ICD-10 codes of fractures) or treated on an outpatient basis using a list of orthopedic splinting procedure codes from the French medical classification of clinical procedures (CCAM) [16].

### Other sociodemographic and health measures

The ESPS also recorded age, sex, country of birth, education level, employment grade, income, marital status, and urbanization level, as well as the European Union Global Activity Limitation Indicator (GALI) and Self-Perceived Health (SPH), recorded in 2010 and 2014 [29]. Mortality (all-cause) evaluated within 5 years of the interview was available from the SNDS database.

### Statistical analysis

#### Prevalence obtained with the indicators

Prevalence estimates were calculated for each of the individual SNDS or ESPS indicators as well as for a composite SNDS indicator (aggregating the three individual SNDS indicators) and another composite SNDS/ESPS indicator using all retrieved cases. A capture-recapture estimate of the prevalence was finally calculated based on the assumption of independence of self-reported and health claims data [30] (an assumption verified both conceptually and operationally). (The capture-recapture method takes advantage of the overlap between self-reported and health claim datasets to estimate the number of cases not retrieved by any of the sources and hence the total number of cases [30].)

#### Agreement between the indicators

Agreement between the (individual and composite) indicators of osteoporosis and fragility fractures was assessed using kappa statistics [31] and 95% confidence intervals (CI), with the latter interpreted in accordance with Landis and Koch: values from 0.0 to 0.20 indicate slight agreement, 0.21 to 0.40 fair agreement, 0.41 to 0.60 moderate agreement, 0.61 to 0.80 substantial agreement, and 0.80 to 1.00 almost perfect agreement [32]. Determinants of disagreement were investigated using polytomous or multinomial logistic regression with adjustments for age and sex, with the cases retrieved from both sources (double positive), which constituted the reference category. (Multinomial logistic regression models the dependency of a nominal outcome [positive for ESPS indicator only, positive for SNDS indicator only, or positive for both indicators] on a set of explanatory variables.) To avoid confusion with the determinants of disease, double negatives (not retrieved from either source, often much more numerous than double positives) were discarded from the analysis.

#### Validity of the indicators

Concurrent validity was evaluated by estimating the associations of osteoporosis and clinical fragility fracture indicators with GALI (any limitation in daily activities vs. no limitation) and SPH (very good and good vs. less than good) in 2010 (baseline). Predictive validity was evaluated by estimating the associations of the indicators with the 2014 health measures (limitation in daily activities and less than good SPH), their evolution (greater limitation, new limitation, SPH deterioration) between 2010 and 2014, and new fragility fractures and death, both within 5 years and assessed using the SNDS. All validity analyses were performed using dichotomous logistic regression providing odds ratios and 95% confidence intervals, adjusted for age, sex, education level, and number of reported comorbidities.

##   Results

The study sample comprised 5039 participants (Table 1), with the majority being women (56%) and aged over 50 years (51%). Limitation in daily activities and less than good SPH were respectively reported by 28% and 37% of the sample at baseline. Mortality and new fragility fractures occurring within 5 years of the 2010 interview affected 5% and 2% of the sample, respectively. Increased limitation in daily activities and deteriorated SPH (in survivors) between 2010 and 2014 concerned 17% and 20% of the sample, respectively.

**Table 1:** Description of the study sample (ESPS 2010 linked to the SNDS and completed health assessment questionnaires, *N* = 5039), *N* and %

	2010 assessment (***N*** = 5039)			
	*N*		%	
Sex, female	2795		56	
*Age*				
25–39 years	1386		28	
40–49 years	1077		21	
50–59 years	1022		20	
60–69 years	738		15	
70–79 years	541		11	
≥ 80 years	275		5	
*Country of birth*				
France	3609		88	
Other	504		12	
Unknown	926			
*Education*				
Less than secondary	1798		36	
Secondary	2062		41	
Tertiary	1179		23	
*Occupation (present or past)*				
Manager, professional	774		15	
Middle manager, teacher	1984		39	
Other, manual worker	2045		41	
No occupation or studying	236		5	
*Employment status*				
Paid employment	2604		52	
Unemployed	452		9	
Homemaker	459		9	
Retired	138		27	
Other	144		3	
*Household income*				
Lower tertile	1571		31	
Middle tertile	1346		27	
Upper tertile	1351		27	
Unknown	771		15	
*Marital status*				
Married/living with a partner	3530		70	
Single/separated/divorced/widowed	1502		30	
Unknown (*N*)	7			
*Urbanization level*				
Less than 2000 inhabitants	1299		26	
2000–19,999 inhabitants	1015		20	
20,000–199,999 habitants	1036		21	
200,000–1,999,999 habitants	1171		23	
City of Paris	518		10	
*Number of chronic conditions reported*				
0	1291		26	
1	1049		21	
2	806		16	
3	616		12	
4 or 5	716		14	
≥ 6	561		11	
	**2010 assessment (*N* = 5039)**		**2014 follow-up (*N* = 2853)**	
	*N*	%	*N*	%
*Self-perceived health*				
Very good–good	3123	63	1561	62
Fair	1399	28	729	29
Bad–very bad	459	9	229	9
Unknown (*N*)	58		334	
Self-perceived health deterioration*				
No			1986	80
Yes			506	20
Unknown (*N*)			361	
*Global activity limitation indicator*				
Not limited	3497	72	1679	68
Limited, not severely	987	20	549	22
Limited, severely	405	8	257	10
Unknown	150		368	
New limitation**				
No			2091	87
Yes			325	14
Unknown (*N*)			437	
Increase in limitation***				
No			1998	83
Yes			418	17
Unknown (*N*)			437	
New fractures within 5 years after 2010 interview****				
No	4916	98		
Yes	123	2		
Deceased within 5 years after 2010 interview****				
No	4807	95		
Yes	232	5		

*ESPS* Health, Health Care, and Insurance Survey, *SNDS* French National Health Data System. ^*^Health graded less in 2014 than in 2010 (in survivors). ^**^Limitation, severe or not in 2014 for those not limited in 2010. ^***^Severe limitation in 2014 for those (survivors) not severely limited in 2010 or any limitation in 2014 for those not limited in 2010. ^****^Assessed in the SNDS

Frequency and prevalence values obtained with the four indicators of osteoporosis and fragility fractures are presented in Table 2. Higher prevalence values were found for self-reported osteoporosis (ESPS) (4.1%), followed by treated osteoporosis (SNDS) (2.2%), clinical fragility fractures (SNDS) (1.2%), and diagnosed osteoporosis (SNDS) (0.4%). With a composite SNDS indicator aggregating diagnosed osteoporosis, treated osteoporosis, and clinical fragility fractures, the prevalence was 3.3%, which was 20% lower than the self-reported indicator (ESPS). Prevalence based on all cases retrieved from either data source (ESPS or SNDS) was 5.6% compared to 7.6% using the capture-recapture estimate of total cases (including those missed by both sources). Supplementary Table 1 presents the characteristics of subjects positive for each of the four indicators, Supplementary Table 2 details the drugs affecting bone structure and mineralization (DABSM) delivered to participants, and Supplementary Table 3 summarizes the sites and severity of clinical fragility fractures (period 2006–2010 and follow-up). Almost all cases of fragility fractures were hospitalized, some of which led to immediate death.

**Table 2:** Frequency and prevalence obtained with the four indicators of osteoporosis

Indicator	Data source	Population considered	Frequency	Prevalence (%) and 95% confidence interval
Self-reported osteoporosis	ESPS	All (≥ 25 years)	207	4.1 (3.6–4.7)
Diagnosed osteoporosis	SNDS	All (≥ 25 years)	21	0.4 (0.2–0.6)
Treated osteoporosis	SNDS	All (≥ 25 years)	110	2.2 (1.8–2.6)
Clinical fragility fracture	SNDS	All (≥ 25 years)	62	1.2 (0.9–1.6)
Clinical fragility fracture	SNDS	≥ 70 years (men) and ≥ 50 years (women)	62	3.5 (2.7–4.5)
Diagnosed or treated osteoporosis or fragility fracture	SNDS	All (≥ 25 years)	165	3.3 (2.8–3.8)
Self-reported, diagnosed, or treated osteoporosis or fragility fractures (all retrieved cases)	EITHER	All (≥ 25 years)	283	5.6 (5.0–6.3)
Self-reported, diagnosed, or treated osteoporosis or fragility fractures (capture-recapture estimate)	EITHER	All (≥ 25 years)	383*	7.6 (6.9–8.4)*

^*^Frequency computed based on the assumption of independent captures between ESPS and SNDS [Hook & Regal]; for prevalence, the interval corresponds to the 95% confidence interval of the estimated total (342–423)

Agreement between the different osteoporosis indicators is shown in Table 3. Agreement was moderate (kappa = 0.48, 95% CI 0.42–0.55) between self-reported and treated osteoporosis. Only 38% of individuals reporting osteoporosis were treated, whereas 72% of those with treated osteoporosis reported the condition. Agreement between all other individual indicators was only slight (*κ* < 0.20). Agreement between clinical fragility fractures and other indicators was especially low, as only 27%, 10%, and 24% of individuals with identified clinical fragility fractures (≥ 70 years for men and ≥ 50 years for women) had self-reported, diagnosed, or treated osteoporosis, respectively. Agreement between self-reported osteoporosis and the SNDS composite indicator was moderate (kappa = 0.46, 95% CI 0.39–0.52): 43% of those reporting osteoporosis were identified in the SNDS, whereas 54% of those identified in the SNDS reported the condition.

**Table 3:** Two-by-two tables of the different indicators of osteoporosis *N* (%) and kappa coefficients and 95% confidence intervals

		Treated osteoporosis (SNDS)			
		Yes	No		
Self-reported osteoporosis	Yes	79 (1.6)	128 (2.5)	207 (4.1)	
	No	31 (0.6)	4801 (95.3)	4832 (95.9)	
		110 (2.2)	4929 (97.8)	5039 (100.0)	Kappa = 0.48 (0.42–0.55)
		Diagnosed osteoporosis (SNDS)			
		Yes	No		
Self-reported osteoporosis	Yes	12 (0.2)	195 (3.9)	207 (4.1)	
	No	9 (0.2)	4823 (95.7)	4832 (95.9)	
		21 (0.4)	5018 (99.6)	5039 (100.0)	Kappa = 0.10 (0.05–0.15)
		Clinical fragility fracture (SNDS, overall population)			
		Yes	No		
Self-reported osteoporosis	Yes	17 (0.3)	190 (3.8)	207 (4.1)	
	No	45 (0.9)	4787 (95.0)	4832 (95.9)	
		62 (1.2)	4977 (98.8)	5039 (100.0)	Kappa = 0.11 (0.06–0.16)
		Clinical fragility fracture (SNDS, at-risk population*)			
		Yes	No		
Self-reported osteoporosis	Yes	17 (0.9)	170 (9.6)	187 (10.6)	
	No	45 (2.6)	1536 (86.9)	1581 (89.4)	
		62 (3.5)	1706 (96.5)	1768 (100.0)	Kappa = 0.09 (0.03–0.15)
		Diagnosed or treated osteoporosis or clinical fragility fracture (SNDS indicators)			
		Yes	No		
Self-reported osteoporosis	Yes	89 (1.8)	118 (2.3)	207 (4.1)	
	No	76 (1.5)	4756 (94.4)	4832 (95.9)	
		165 (3.3)	4874 (96.7)	5039 (100.0)	Kappa = 0.46 (0.39–0.52)
		Treated osteoporosis (SNDS)			
		Yes	No		
Clinical fragility fracture (SNDS, overall population)	Yes	15 (0.3)	47 (0.9)	62 (1.2)	
	No	95 (1.9)	4882 (96.9)	4977 (98.8)	
		110 (2.2)	4929 (97.8)	5039 (100.0)	Kappa = 0.16 (0.08–0.24)
		Treated osteoporosis (SNDS)			
		Yes	No		
Clinical fragility fracture (SNDS, overall population)	Yes	15 (0.8)	47 (2.7)	62 (3.5)	
	No	88 (5.0)	1618 (91.5)	1706 (96.5)	
		103 (5.8)	1665 (94.2)	1768 (100.0)	Kappa = 0.14 (0.06–0.23)
		Treated osteoporosis (SNDS)			
		Yes	No		
Clinical fragility fracture (SNDS, at-risk population*, male)	Yes	2 (0.5)	12 (3.1)	14 (3.6)	
	No	8 (2.1)	363 (94.3)	371 (96.4)	
		10 (2.6)	375 (97.4)	385 (100.0)	Kappa = 0.14 (0.00–0.35)
		Treated osteoporosis (SNDS)			
		Yes	No		
Clinical fragility fracture (SNDS, at-risk population*, female)	Yes	13 (0.9)	35 (2.6)	48 (3.5)	
	No	80 (5.8)	1255 (90.7)	1335 (96.5)	
		93 (6.7)	1290 (93.3)	1383 (100.0)	Kappa = 0.15 (0.06–0.23)
		Diagnosed osteoporosis (SNDS)			
		Yes	No		
Treated osteoporosis (SNDS)	Yes	9 (0.2)	101 (2.0)	110 (2.2)	
	No	12 (0.2)	4917 (97.6)	4929 (97.8)	
		21 (0.4)	5018 (99.6)	5039 (100.0)	Kappa = 0.13 (0.05–0.21)
		Diagnosed osteoporosis (SNDS)			
		Yes	No		
Clinical fragility fracture (overall population)	Yes	6 (0.1)	56 (1.1)	62 (1.2)	
	No	15 (0.3)	4962 (98.5)	4977 (98.8)	
		21 (0.4)	5018 (99.6)	5039 (100.0)	Kappa = 0.14 (0.04–0.24)
		Diagnosed osteoporosis (SNDS)			
		Yes	No		
Clinical fragility fracture (at-risk population*)	Yes	6 (0.3)	56 (3.2)	62 (3.5)	
	No	14 (0.8)	1692 (95.7)	1706 (96.5)	
		20 (1.1)	1748 (97.9)	1768 (100.0)	Kappa = 0.13 (0.03–0.24)

^*^At-risk population: ≥ 70 years (men) and ≥ 50 years (women), *N* = 1768 including 385 men and 1383 women

Supplementary Table 4 presents the factors associated with each disagreement type for all the pairs of indicators except for diagnosed osteoporosis (due to the small sample size). Only a few significant associations emerged: secondary or tertiary education was associated with lower odds of being treated without reporting osteoporosis but higher odds of being treated without having a clinical fragility fracture. The number of chronic conditions (≥ 3) was associated with lower odds of having a fragility fracture without reporting osteoporosis. Limitation in daily activities was associated with higher odds of being treated without reporting osteoporosis but higher odds of fracture without reporting osteoporosis.

Concurrent and predictive validity results are presented in Table 4. Despite often-overlapping confidence intervals of odds ratios, diagnosed osteoporosis, treated osteoporosis, and clinical fragility fractures were more consistently and strongly associated with 2010 and 2014 health measures and their evolution along with 5-year mortality compared to self-reported osteoporosis. Diagnosed osteoporosis and clinical fragility fractures in 2010 were especially predictive of new fractures (odds ratio = 5.54, 95% CI 2.07–14.82 and odds ratio = 2.95, 95% CI 1.51–5.74, respectively). The composite SNDS indicator had the best concurrent and predictive validity overall, clearly outperforming the self-reported indicator in terms of predicting new fractures (odds ratio = 2.57, 95% CI 1.59–4.16) and almost all other health outcomes.

**Table 4:** Association between ESPS and SNDS indicators of osteoporosis with health outcomes. Odds ratios and 95% confidence intervals adjusted for age, sex, education, and number of chronic conditions (≥ 2 vs. < 2)*

	Self-reported osteoporosis	Diagnosed osteoporosis (SNDS)	Treated osteoporosis (SNDS)	Clinical fragility fracture (SNDS)	Diagnosed or treated osteoporosis or clinical fragility fracture (SNDS)
***Concurrent validity***					
*2010 self-perceived health*					
Very good–good	1.00	**1.00**	1.00	1.00	**1.00**
Fair-bad–very bad	**1.51 (1.09–2.08)**	**4.28 (1.21–15.10)**	**1.77 (1.22–2.57)**	1.41 (0.80–2.49)	**1.93 (1.33–2.80)**
*2010 global activity limitation indicator*					
Not limited	1.00	**1.00**	1.00	1.00	**1.00**
Limited, any severity	1.26 (0.93–1.71)	**3.92 (1.41–10.91)**	**2.02 (1.41–2.88)**	**2.37 (1.35–4.16)**	**2.20 (1.54–3.13)**
***Predictive validity***					
*New fracture within 5 years after 2010 interview*					
No	1.00	**1.00**	1.00	1.00	1.00
Yes	1.53 (0.90–2.57)	**5.54 (2.07–14.82)**	1.48 (0.87–2.50)	**2.95 (1.51–5.74)**	**2.57 (1.59–4.16)**
*Death within 5 years after 2010 interview*					
No	1.00	1.00	1.00	**1.00**	**1.00**
Yes	0.89 (0.51–1.55)	1.76 (0.49–6.29)	**1.73 (1.05–2.85)**	**2.29 (1.17–4.49)**	**1.67 (1.01–2.76)**
*2014 self-perceived health*					
Very good–Good	1.00	1.00	1.00	1.00	1.00
Fair-bad–very bad	**1.51 (1.00–2.29)**	8.54 (0.99–73.91)	**1.86 (1.10–3.14)**	1.03 (0.46–2.28)	**1.89 (1.12–3.17)**
Perceived health deterioration**					
No	1.00	1.00	1.00	1.00	1.00
Yes	1.42 (0.89–2.28)	-	0.52 (0.25–1.10)	0.32 (0.08–1.36)	0.52 (0.24–1.09)
*2014 global activity limitation indicator*					
Not limited	1.00	**1.00**	1.00	1.00	**1.00**
Limited, any severity	**1.61 (1.07–2.41)**	**10.08 (1.18–86.29)**	**1.73 (1.06–2.82)**	1.34 (0.62–2.88)	**1.87 (1.14–3.04)**
Increase in limitation**					
No	1.00	1.00	1.00	1.00	1.00
Yes	1.39 (0.88–2.18)	3.19 (0.70–14.65)	1.57 (0.93–2.62)	1.47 (0.67–3.21)	1.56 (0.93–2.62)
New limitation**					
No	1.00	1.00	1.00	1.00	1.00
Yes	1.38 (0.84–2.27)	0.92 (0.11–7.92)	1.09 (0.59–2.01)	1.12 (0.45–2.79)	1.09 (0.59–2.00)

Bold characters indicate statistically significant associations

*ESPS* Health, Health Care, and Insurance Survey, *SNDS* French National Health Data System. ^*^Categories as in Table 1 except for chronic conditions in two categories (≥ 2 vs. < 2). ^**^Defined as in Table 1

## Discussion

Taking advantage of the linkage between a large nationwide health interview survey with follow-up (ESPS) and the French National Health Data System (SNDS), this study evaluated the agreement and comparative validity (concurrent and predictive) of four indicators relating to osteoporosis and fragility fractures. Agreement between indicators was only slight to fair, although it was occasionally moderate with regard to education level, limitation in daily activities, and number of chronic conditions. SNDS indicators displayed better concurrent and predictive validity than the self-reported indicator, especially in terms of predicting new fractures. A composite indicator built with the primary SNDS indicators showed the best concurrent and predictive validity but only moderate concordance with self-reported osteoporosis. It should therefore be coupled with self-reported data to better assess the burden of the condition.

Substantial discordance was found between self-reported and treated osteoporosis, which is easily traceable in healthcare claims databases, with a kappa coefficient of 0.48, close to that reported by Peeters et al. in Australia (0.51) [21] but lower than that reported in glucocorticoid users in the USA (0.64) [33]. Discordance was observed in both directions, since only 38% of individuals reporting osteoporosis were treated, whereas only 72% of those with treated osteoporosis reported the condition. This discrepancy suggests deficiencies in the information provided by prescribers, which is all the more worrying given that the treatments considered in this study (DABSM, excluding calcium and/or vitamin D) are specific to osteoporosis. Nevertheless, this proportion was similar to the study of Peeters et al. [21], although the authors did not provide the medication names or codes. Self-reported osteoporosis was also discordant with LBMD in two studies on this topic (with kappas of 0.24 [23] and 0.29[22]). One of these studies addressed the factors associated with disagreement, showing that risk factors for osteoporosis (i.e., old age, female sex, non-Black race, low BMI, history of fractures, and treatment) were also factors of disagreement [23], although it did not discard double negatives. Our analysis of the factors associated with disagreement between self-reported and treated osteoporosis only identified education level and limitation in daily activities as positively associated with treatment without self-reported osteoporosis (i.e., “uninformed osteoporotic subjects”).

**Fig. 1:**
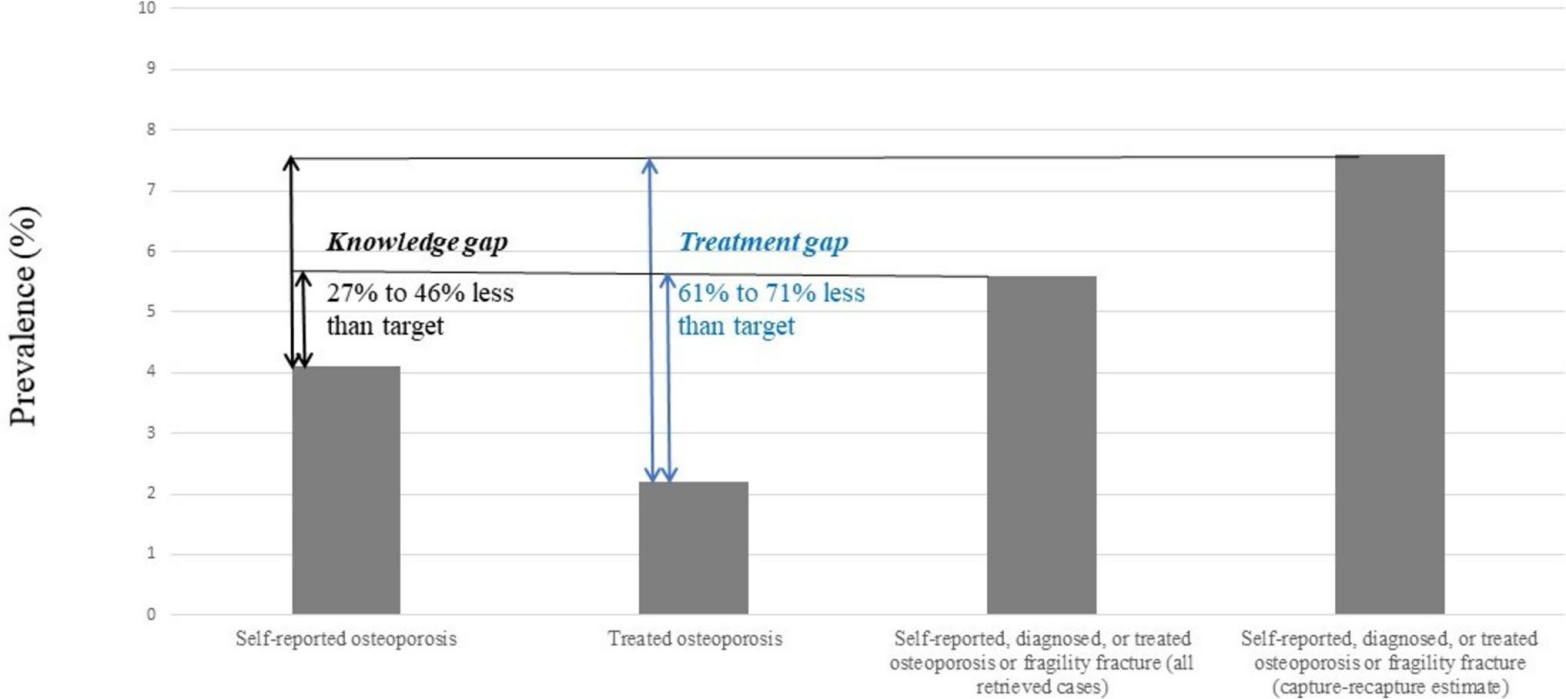
Knowledge and treatment gaps for subjects identified with osteoporosis or fragility fractures in France. Percentages in the population

Similar to osteoporosis treatments, clinical fragility fractures, at least those hospitalized or specifically treated, are easily traceable in healthcare claims databases [34]. In our study, the agreement between clinical fragility fractures (defined as every fracture occurring ≥ 70 years for men and ≥ 50 years for women) and other indicators was very low: kappas were well below 0.20, and only 27% and 24% of individuals with identified clinical fragility fractures also reported osteoporosis or were treated. Only 10% of subjects with identified clinical fragility fractures had diagnosed osteoporosis, although diagnosis was limited to ICD-10 codes for LTDs and inpatients. When considering all cases retrieved by any indicator and especially total cases including those missed by both the SNDS and ESPS, as permitted by the capture-recapture method [30], the “knowledge gap” (defined here as the relative difference between self-reported and identified cases) and the “treatment gap” (defined here as the relative difference between treated and identified cases) were estimated to range from 27 to 46% and from 61 to 71%, respectively (Fig. 1). The latter figure is just below the value of 82% recently estimated in France using the FRAX method to identify osteoporotic subjects [35].

As expected, healthcare claims data-derived indicators of osteoporosis (reflecting actual care provided) were more strongly associated with health outcomes (perceived health, limitation, and mortality [36]) and especially the occurrence of new fractures compared to self-reported osteoporosis. Diagnosed osteoporosis and clinical fragility fractures were found to be strongly predictive of new fractures, although the former indicator was much less sensitive than the latter, with only 0.4% of subjects identified versus 1.2% for the latter. Aggregating diagnosed osteoporosis, treated osteoporosis, and clinical fragility fractures produced a single composite SNDS indicator with far better concurrent and predictive validity than self-reported osteoporosis, although its sensitivity was still low, since 42% and 57% of cases respectively retrieved from either source or estimated using the capture-recapture method were not identified by the composite indicator. It is regrettable that diagnoses from outpatient consultations are not coded in France as routinely done in several countries such as Canada. Indeed, a Canadian healthcare claims database algorithm identifying osteoporosis (with hospital diagnosis, physician diagnosis, and osteoporosis treatment within the past year) was validated against LBMD [20]. Note, however, that the lack of sensitivity of several health claims-based indicators (especially the “diagnosed osteoporosis” indicator) has no impact on the prevalence estimate provided by the capture-recapture method or on the derived estimates of knowledge and treatment gap.

This study has many strengths: its nationwide representative sample, the health interview survey with follow-up and prospective assessment of health outcomes, mortality, and clinical fragility fractures, its linkage to a large healthcare claims database, and an adequate approach to identify disagreements between data sources. However, several features of the data sources should be taken into account when interpreting the findings. First, and as already mentioned, the SNDS database does not contain the codes for outpatient consultations. This inadequacy is partly offset by the coding of LTDs, a system that allows patients with chronic diseases requiring long-term and/or costly care to benefit from free healthcare, although this system is rarely used for osteoporotic or fractured subjects. Second, due to the insufficient quality of data for several health insurance regimes during the period 2006–2010, this study was restricted to subjects affiliated to the general scheme and local mutualist sections of the French National Health Insurance (87% of the population), notably excluding independent workers and farmers. Further, the study was limited to survey participants living in the community who accepted to complete the health questionnaires, meaning that the findings may not be generalizable to the overall French population. Third, the data analyzed is relatively outdated (2010–2015) and does not include recent DABSM. However, the ESPS is the last large general population survey conducted in France that could be linked to the SNDS. Regarding the prevalence estimations, even though our data may seem dated, previous data on self-reported osteoporosis and fractures in the population date back 20 years [10]. Nevertheless, a decrease in DABSM prescriptions since 2010 has been reported in France [37].

The time has come to diversify methods used to assess the burden of osteoporosis and fragility fractures in the general population. Healthcare claims databases, already used for surveillance purposes for various conditions, may also be relevant for monitoring osteoporosis. In France, the SNDS provides valid but far from sufficient indicators for assessing and monitoring this burden. Coupling these indicators with self-reported data may overcome some of their limitations and highlight the treatment, knowledge, and information “gaps” associated with the condition. Among the indicators derived from healthcare claims databases, clinical fragility fractures, which have indisputable validity [38] and are easily traceable in claims databases, should probably play a central role in epidemiological surveillance due to their direct association with mortality [39]. In the case of a surveillance system linked to secondary prevention policies, the occurrence of the first fragility fracture may represent a timely opportunity for personalized interventions. An example of such a policy is the “Capture the Fracture® Best Practice Framework” implemented by the International Osteoporosis Foundation [40] in 61 countries (including France, with the participation of 33 “Fracture Liaison Services”). This approach is complementary to the primary prevention methods that can avoid or delay the occurrence of the first fragility fracture.

## Supplementary Information

Below is the link to the electronic supplementary material.

ESM1 (PDF 199 KB)

## Data Availability

The data that support the findings of this study are available from the corresponding author upon reasonable request.
