# Supplementary material for: Assessing the burden of osteoporosis and clinical fragility fractures in the French general population: insights from linked healthcare claims and health interview survey data used for surveillance
Source: Arch Osteoporos. 2025 Oct 27;20(1):136. doi: 10.1007/s11657-025-01616-2 (PMC12559036; doi:10.1007/s11657-025-01616-2)
Supplement: Supplementary file 1 — (PDF 199 KB) [file 11657_2025_1616_MOESM1_ESM.pdf]

# Assessing the burden of osteoporosis and clinical fragility fractures in the French general population: Insights from linked healthcare claims and health interview survey data used for surveillance

## Supplementary material

Supplementary Figure 1. Flowchart of the study

Supplementary Table 1. Characteristics of participants according to whether they 1) self-reported osteoporosis (ESPS), 2) had received a diagnosis of osteoporosis (SNDS), 3) were treated for osteoporosis (SNDS), and 4) had a clinical fragility fracture (SNDS)

Supplementary Table 2. Supplementary Table 2. Drugs affecting bone structure and mineralization (DABSM) delivered to participants within the past year (N=110)

Supplementary Table 3. Sites and severity of clinical fragility fractures identified in the SNDS

Supplementary Table 4. Sociodemographic and health characteristics associated with each discordant group compared with the concordant group for pairs of indicators among self-reported osteoporosis, treated osteoporosis, and fragility fractures (diagnosed osteoporosis [SNDS] was not considered due to the limited sample size of this group)

Supplementary Figure 1. Flowchart of the study

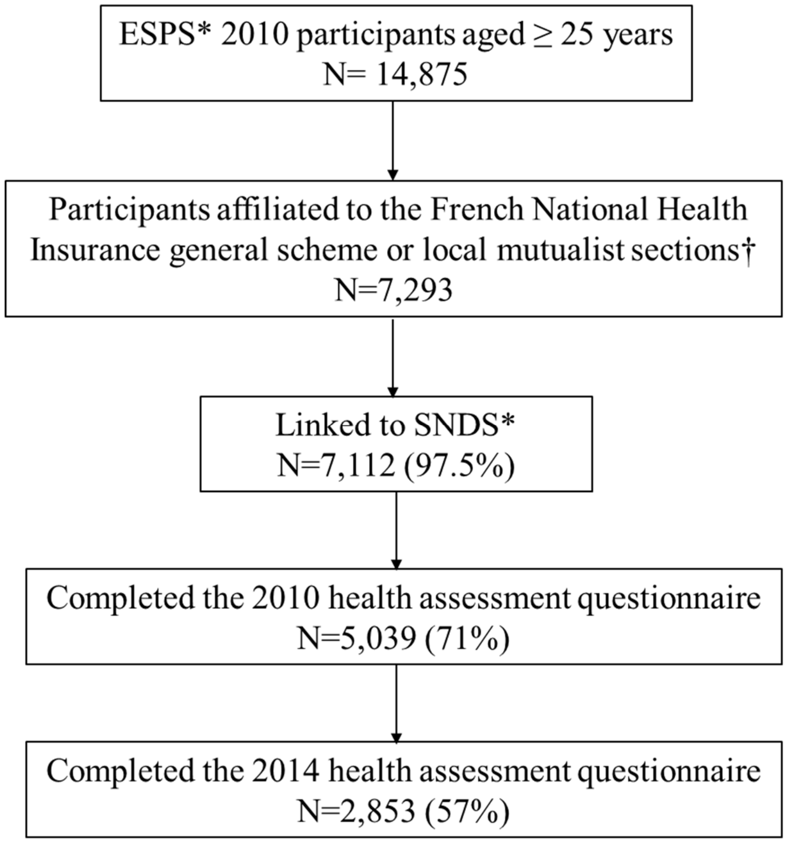


* ESPS: Health, Health Care, and Insurance Survey; SNDS: French National Health Data System

† The general scheme covers salaried employees working in the private sector and their dependents (i.e., about 76% of the population), while the local mutualist sections cover civil servants, local government employees, public hospital workers, and students (i.e., about 11% of the population)

Supplementary Table 1. Characteristics of participants according to whether they 1) self-reported osteoporosis (ESPS), 2) had received a diagnosis of osteoporosis (SNDS), 3) were treated for osteoporosis (SNDS), and 4) had a clinical fragility fracture (SNDS)

| | Self-reported osteoporosis (N=207) | | Diagnosed osteoporosis (N=21) | | Treated osteoporosis (N=110) | | Clinical fragility fracture (N=62) | |
|---|---|---|---|---|---|---|---|---|
| | N | % | N | % | N | % | N | % |
| Sex, female | 186 | 90 | 18 | 86 | 96 | 87 | 48 | 77 |
| *Age* | | | | | | | | |
| 25-39 years | 3 | 1 | 0 | 0 | 2 | 2 | 0 | 0 |
| 40-49 years | 6 | 3 | 0 | 0 | 1 | 1 | 0 | 0 |
| 50-59 years | 30 | 14 | 4 | 19 | 13 | 12 | 8 | 13 |
| 60-69 years | 77 | 37 | 4 | 19 | 30 | 27 | 17 | 27 |
| 70-79 years | 57 | 28 | 7 | 33 | 35 | 32 | 22 | 36 |
| ≥ 80 years | 34 | 16 | 6 | 29 | 29 | 26 | 15 | 24 |
| *Country of birth* | | | | | | | | |
| France | 153 | 87 | 15 | 83 | 79 | 84 | 45 | 88 |
| Other | 23 | 13 | 3 | 17 | 15 | 16 | 6 | 12 |
| Unknown | 31 | | 3 | | 16 | | 11 | |
| *Education* | | | | | | | | |
| Less than secondary | 99 | 48 | 10 | 48 | 60 | 55 | 40 | 65 |
| Secondary | 76 | 37 | 7 | 33 | 30 | 27 | 15 | 24 |
| Tertiary | 32 | 15 | 4 | 19 | 20 | 18 | 7 | 11 |
| *Occupation (present or past)* | | | | | | | | |
| Manager, professional | 28 | 14 | 4 | 19 | 19 | 17 | 13 | 21 |
| Middle manager, teacher | 108 | 52 | 6 | 29 | 56 | 51 | 23 | 37 |
| Other, manual worker | 57 | 28 | 10 | 48 | 24 | 22 | 23 | 37 |
| No occupation or studying | 14 | 7 | 1 | 5 | 11 | 10 | 3 | 5 |
| *Employment status* | | | | | | | | |
| Paid employment | 24 | 12 | 0 | 0 | 13 | 12 | 5 | 8 |
| Unemployed | 5 | 2 | 0 | 0 | 0 | 0 | 2 | 3 |
| Homemaker | 20 | 10 | 2 | 10 | 16 | 15 | 7 | 11 |
| Retired | 146 | 71 | 16 | 76 | 77 | 70 | 47 | 76 |
| Other | 12 | 6 | 3 | 14 | 4 | 4 | 1 | 2 |
| *Household income* | | | | | | | | |
| Lower tertile | 77 | 37 | 11 | 52 | 38 | 35 | 25 | 40 |
| Middle tertile | 50 | 24 | 5 | 24 | 25 | 23 | 9 | 15 |
| Upper tertile | 29 | 14 | 2 | 10 | 15 | 14 | 10 | 16 |
| Unknown | 51 | 25 | 3 | 14 | 32 | 29 | 18 | 29 |
| *Marital status* | | | | | | | | |
| Married/living with a partner | 124 | 60 | 14 | 67 | 66 | 60 | 35 | 56 |
| Single/separated/divorced/widov | 83 | 40 | 7 | 33 | 44 | 40 | 27 | 44 |
| Unknown (N) | 0 | | | | 0 | | | |
| *Urbanization level* | | | | | | | | |
| Less than 2,000 inhabitants | 34 | 16 | 3 | 14 | 25 | 23 | 7 | 11 |
| 2,000-19,999 inhabitants | 45 | 22 | 3 | 14 | 24 | 22 | 14 | 22 |
| 20,000-199,999 habitants | 55 | 27 | 6 | 29 | 30 | 27 | 19 | 31 |
| 200,000- 1,999,999 habitants | 50 | 24 | 7 | 33 | 23 | 21 | 16 | 26 |
| City of Paris | 23 | 11 | 2 | 10 | 8 | 7 | 6 | 10 |
| *Number of chronic conditions reported* | | | | | | | | |
| 0 | 0 | 0 | 0 | 0 | 1 | 1 | 3 | 5 |
| 1 | 14 | 7 | 2 | 10 | 12 | 11 | 6 | 9 |
| 2 | 19 | 9 | 5 | 24 | 12 | 11 | 9 | 15 |
| 3 | 35 | 17 | 2 | 10 | 18 | 16 | 9 | 15 |
| 4 or 5 | 41 | 20 | 0 | 0 | 29 | 26 | 15 | 24 |
| ≥ 6 | 98 | 47 | 12 | 57 | 38 | 35 | 20 | 32 |

ESPS: Health, Health Care, and Insurance Survey; SNDS: French National Health Data System

Supplementary Table 2. Drugs affecting bone structure and mineralization (DABSM) delivered to participants within the past year (N=110). Name and Anatomical Therapeutic Chemical (ATC) codes

| Name | ATC code | N |
|---|---|---|
| Etidronic acid | M05BA01 | 0 |
| Clodronic acid | M05BA02 | 1 |
| Alendronic acid | M05BA04 | 15 |
| Ibandronic acid | M05BA06 | 22 |
| Risedronic acid | M05BA07 | 23 |
| Zoledronic acid | M05BA08 | 1 |
| Alendronic acid and colecalciferol | M05BB03 | 18 |
| Risedronic acid, calcium and colecalciferol, sequential | M05BB04 | 1 |
| Strontium ranelate | M05BX03 | 14 |
| Several classes | – | 15 |

Supplementary Table 3. Sites and severity of clinical fragility fractures identified in the SNDS*

| | 2006-2010 fractures (N=62) | Follow-up fractures** (N=123) |
|---|---|---|
| *Site* | | |
| Proximal upper limb | 7 | 12 |
| Distal upper limb | 21 | 24 |
| Proximal lower limb | 10 | 43 |
| *including femoral neck* | 10 | 39 |
| Distal lower limb | 15 | 21 |
| Other | 11 | 31 |
| *Severity* | | |
| Hospitalization | 58 | 120 |
| Death (during hospital stay) | 0 | 3 |

* Total exceeds 62 or 123, since participants can have fractures at multiple sites
** Fractures occurring within 5 years after 2010 interview

Supplementary Table 4. Sociodemographic and health characteristics associated with each discordant group compared with the concordant group for pairs of indicators among self-reported osteoporosis, treated osteoporosis, and fragility fractures (diagnosed osteoporosis [SNDS] was not considered due to the limited sample size of this group). Odds ratios and 95% confidence intervals estimated by polytomic logistic regression, including sex as a covariate.

| | Self-reported osteoporosis vs. treated osteoporosis (SNDS) | | Self-reported osteoporosis vs. clinical fragility fracture (SNDS) | | Treated osteoporosis (SNDS) vs. clinical fragility fracture (SNDS) | |
|---|---|---|---|---|---|---|
| | Self-reported only | Treated only | Self-reported only | Fracture only | Treated only | Fracture only |
| *Sex* | | | | | | |
| Male | 1.00 | 1.00 | 1.00 | 1.00 | 1.00 | 1.00 |
| Female | 0.79 (0.30–2.06) | 0.34 (0.11–1.06) | 0.53 (0.07–4.22) | 0.15 (0.02–1.28) | 1.07 (0.21–5.31) | 0.45 (0.09–2.28) |
| *Age* | | | | | | |
| <60 years | 1.52 (0.69–3.31) | 1.52 (0.45–5.05) | 0,29 (0.04–2.29) | 0.32 (0.04–2.95) | 0.41 (0.04–3.54) | 0.42 (0.04–3.88) |
| 60-79 years | 1.00 | 1.00 | 1.00 | 1.00 | 1.00 | 1.00 |
| ≥ 80 years | 0.53 (0.25–1.13) | 2.04 (0.79–5.25) | 0.15 (0.02–1.36) | 0.26 (0.03–2.81) | 0.31 (0.03–3.03) | 0.24 (0.02–2.58) |
| *Education* | | | | | | |
| Less than secondary or Unknown | 1.00 | 1.00 | 1.00 | 1.00 | 1.00 | 1.00 |
| Secondary or Tertiary | 0.87 (0.49–1.57) | **0.39 (0.16–0.99)*** | 2.81 (0.95–8.29) | 1.36 (0.40–4.61) | **6.75 (1.44–31.68)*** | 4.56 (0.92–22.68) |
| *Occupation (present or past)* | | | | | | |
| Manager, professional | 1.00 | 1.00 | 1.00 | 1.00 | 1.00 | 1.00 |
| Others | 1.39 (0.62–3.12) | 0.85 (0.29–2.51) | 0.34 (0.04–2.70) | 0.31 (0.03–2.83) | 0.49 (0.06–4.13) | 0.43 (0.05–3.94) |
| *Employment status* | | | | | | |
| Paid employment or Unemployed | 1.00 | 1.00 | 1.00 | 1.00 | 1.00 | 1.00 |
| Others | 1.32 (0.49–3.58) | 1.61 (0.31–8.31) | 1.56 (0.41–5.94) | 1.09 (0.25–4.82) | 1.24 (0.29–5.23) | 1.22 (0.26–5.67) |
| *Household income* | | | | | | |
| Lower or Middle third tertile | 1.00 | 1.00 | 1.00 | 1.00 | 1.00 | 1.00 |
| Upper third tertile or Unknown | 0.74 (0.41–1.32) | 0.98 (0.42–2.28) | 1.13 (0.40–3.21) | 1.52 (0.47–4.89) | 0.83 (0.28–2.52) | 0.82 (0.25–2.70) |
| *Marital status* | | | | | | |
| Married/living with a partner | 1.00 | 1.00 | 1.00 | 1.00 | 1.00 | 1.00 |
| Single/separated/divorced/widowed | 1 .37 (0.75–2.51) | 1.38 (0.57–3.33) | 0.77 (0.28–2.10) | 1.09 (0.34–3.46) | 0.72 (0.24–2.18) | 0.95 (0.29–3.12) |
| *Urbanization level* | | | | | | |
| Less than 200,000 inhabitants | 1.00 | 1.00 | 1.00 | 1.00 | 1.00 | 1.00 |
| More than 200,000 inhabitants | 1.10 (0.61–1.96) | 0.46 (0.19–1.07) | 0.48 (0.15–1.52) | 0.54 (0.15–1.96) | 0.40 (0.12–1.36) | 0.63 (0.17–2.31) |
| *Number of chronic conditions reported* | | | | | | |
| < 3 | 1.00 | 1.00 | 1.00 | 1.00 | 1.00 | 1.00 |
| ≥ 3 | 1.74 (0.80–3.76) | 0.36 (0.13 - 1.01) | 0.31 (0.04–2.43) | **0.11 (0.01–0.88)**** | 0.48 (0.10–2.29) | 0.30 (0.06–1.52) |
| *Self-perceived health* | | | | | | |
| Very good - Good | 1.00 | 1.00 | 1.00 | 1.00 | 1.00 | 1.00 |
| Less than good | 1.00 (0.54–1.84) | 1.83 (0.65–5.12) | 1.03 (0.36–2.92) | 0.90 (0.28–2.93) | 0.83 (0.24–2.84) | 0.56 (0.15–2.04) |
| *Global Activity Limitation Indicator* | | | | | | |
| Not limited | 1.00 | 1.00 | 1.00 | **1.00** | 1.00 | 1.00 |
| Limited, any severity | 1.20 (0.66–2.19) | **2.97 (1.10–8.03)*** | 1.45 (0.53–4.00) | **3.30 (1.01–10.80)*** | 1.14 (0.37–3.51) | 1.64 (0.49–5.52) |

ESPS: Health, Health Care, and Insurance Survey; SNDS: French National Health Data System
*: p<0.05 ; **: p<0.01 ; ***: p<0.001
